# Virtual reality as classification aid for acetabular fractures: New possibilities for training and teaching

**DOI:** 10.1007/s00113-026-01705-y

**Published:** 2026-04-01

**Authors:** Vincent K. Schenk, Carolin Dieges, David Ackermann, Maximilian M. Menger, Carolina Vogel, Tina Histing, Steven C. Herath, Markus A. Küper, Anna L. Schiltenwolf, Christof K. Audretsch

**Affiliations:** 1 Department for Traumatology and Reconstructive Surgery, BG Trauma Center, University of Tübingen, Schnarrenbergstraße 95, 72076 Tübingen, Germany; 2 Faculty of Medicine, Eberhard Karls University of Tübingen, Tübingen, Germany

**Keywords:** Virtual reality, Acetabular surgery, Fracture classification, Medical education, Medical simulation, Virtual Reality, Acetabulumchirurgie, Frakturklassifikation, Medizinische Ausbildung, Medizinische Simulation

## Abstract

**Background:**

The incidence of acetabular fractures has increased, and classification according to Judet and Letournel remains challenging. While 3D CT improves accuracy, virtual reality (VR) may enhance training. This study assessed whether a VR simulator improves classification accuracy and understanding in inexperienced users.

**Materials and methods:**

An HTC VIVE Pro headset and an Alienware m15 R4 computer were utilized. Programming was done in Unity (2021.3.4f1 LTS), and segmentation in Slicer3D. In total, 83 ninth-semester medical students, divided into a VR group (*n* = 44) and a 3D control group (*n* = 39), took part. Participants had to classify 11 acetabular fractures, supported by pictograms if needed, followed by a user survey. Accuracy was compared.

**Results:**

All participants completed the study. Overall, 68% had no prior knowledge of the classification. The control group achieved a median of 27% (interquartile range [IQR]: 14–36%) correct classifications, while the VR group reached 45% (IQR: 36–64%), showing higher accuracy (*p* < 0.001). In general, essential fractures were classified significantly more accurately than associated fractures (*p* < 0.001). The VR system was rated as more intuitive than the 3D software (*p* = 0.025).

**Conclusion:**

The VR simulator significantly improved acetabular fracture classification among inexperienced users. It is intuitive, easy to use, and offers strong potential for future surgical training.

**Graphic abstract:**

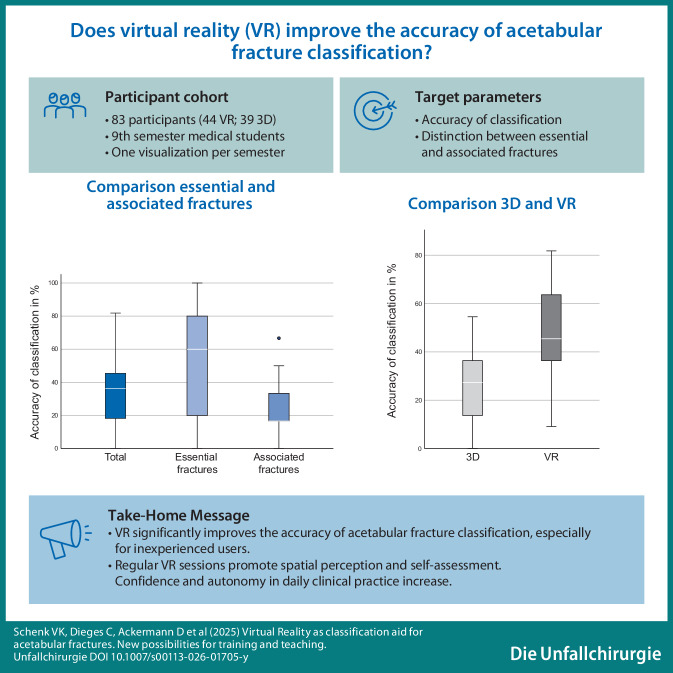

## Abstract (de)

**Hintergrund:**

Die Inzidenz von Acetabulumfrakturen steigt, die Klassifikation nach Judet und Letournel bleibt jedoch herausfordernd. Während die 3‑D-Computertomographie (CT) die Genauigkeit verbessert, könnte Virtual Reality (VR) das Training unterstützen. Ziel dieser Studie war die Evaluation eines VR-Simulators hinsichtlich Verständnis und Klassifikationsgenauigkeit.

**Material und Methoden:**

Ein HTC-VIVE-Pro-Headset und ein Alienware-m15-R4-Computer wurden verwendet. Die Entwicklung erfolgte in Unity (2021.3.4f1 LTS), die Segmentierung in Slicer3D. Getestet wurde das System von 83 Studierenden des 9. Semesters, aufgeteilt in eine VR-Gruppe (*n* = 44) und eine 3‑D-Kontrollgruppe (*n* = 39). Jede Person klassifizierte 11 Acetabulumfrakturen, unterstützt durch Piktogramme. Anschließend erfolgten die Auswertung und eine Befragung.

**Ergebnisse:**

Alle Teilnehmenden schlossen die Studie ab. 68 % gaben keine Vorkenntnisse an. Die Kontrollgruppe erreichte im Median 27 % (Interquartilsabstand, IQR: 14–36 %), die VR-Gruppe 45 % (IQR 36–64 %; *p* < 0,001). Generell wurden einfache Frakturen signifikant häufiger korrekt klassifiziert als komplexe (*p* < 0,001). Das VR-System wurde als signifikant intuitiver bewertet als die 3‑D-Software (*p* = 0,025).

**Schlussfolgerung:**

Der VR-Trainingssimulator verbesserte die Klassifikation von Acetabulumfrakturen bei unerfahrenen Anwendern signifikant. Er ist intuitiv, benutzerfreundlich und zeigt großes Potenzial für die chirurgische Aus- und Weiterbildung.

## Introduction

The number of acetabular fractures has risen significantly in recent years, particularly among older patients with osteoporosis. For young surgeons and medical students, the complex pelvic anatomy and diverse fracture patterns make the Judet and Letournel classification challenging to master. Virtual reality (VR) offers an innovative, immersive training approach to improve understanding and classification accuracy.

Although still rare compared to other trauma cases, acetabular fractures (AF) have increased markedly, rising by 58% between 2009 and 2019 due to an aging population [1–3]. Incidence rates of 4.3 per 100,000 in patients under 70 and 43.9 per 100,000 in those over 70 have been reported [2], while rates in younger individuals remain stable [1, 3]. High-energy trauma predominates in young patients, whereas low-energy osteoporotic falls are typical in older adults [1], increasingly contributing to morbidity with rising life expectancy [4, 5].

Letournel and Judet first systematically analyzed AFs, establishing a classification of ten fracture types, which remains the gold standard today. Five essential fractures, involving a single fracture line, and five associated fractures, consisting of combinations of essential fracture types, can be differentiated. The classification guides surgical management and affects patient outcomes [6, 7]. Accuracy strongly correlates with surgical experience [8]; experts show moderate agreement, whereas novices achieve correct classifications in only about 40% of cases, especially with plain radiographs [9, 10]. Limited case exposure and complex 3D anatomy present additional challenges for training [11].

Virtual reality (VR) provides an immersive, reproducible learning environment using a head-mounted display that translates user movements into a simulated setting [12]. In surgical education, VR supports structured skill acquisition as clinical training opportunities decline [11, 13–16]. Additionally, studies show an improvement in performance and learning efficiency in trauma surgery [13, 16, 17].

Based on this, we developed a VR tool to enhance understanding and classification of AFs and evaluated whether immersive VR improves comprehension and accuracy among inexperienced users.

## Materials and methods

### Materials

An HTC VIVE Pro (HTC Corporation, New Taipei City, Taiwan) headset with hand controllers and Lighthouse tracking was used, connected to a VR-ready computer (Alienware m15 R4; Dell Inc., Round Rock, TX, USA). The simulator was developed in Unity (v2021.3.3f1 LTS) using C# with SteamVR and VIVE Input Utility plugins. A total of 11 anonymized pelvic models from patient computed tomography (CT) scans from our institution were segmented semi-automatically in 3D Slicer and integrated into the program [18, 19].

### Methods

#### 3D visualization tool

**Fig. 1:**
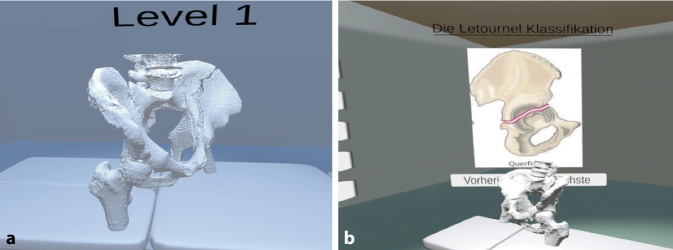
In-game view of the virtual operating room in both classification programs. **a** 3D classification program with 40° view of the pelvis. **b** Virtual reality classification program with slideshow of classification pictograms

We aimed to develop a CT-comparable visualization tool with enhanced rotational control. The Unity-based Windows application displays the pelvis on an operating table, enabling free gaze movement and 20° rotations via the spacebar (Fig. 1a). User position is fixed. Letournel classification is accessible via the numeric keypad, with printed pictograms available for reference.

#### VR simulator

The Unity-based VR simulator enables free navigation in a virtual operating room with unrestricted pelvic views. Letournel pictograms are provided for comparison, and classifications are selected via a virtual laser before advancing to the next level (Fig. 1b).

#### Participants

The VR simulator was evaluated in a cohort of ninth-semester medical students over two semesters. Students in the first semester served as the 3D control group, while those in the second semester used the VR simulator. Participation was voluntary. Differences in student enrollment each semester resulted in unequal group sizes. All students had access to the same optional lecture on pelvic and AFs two semesters earlier, which was also part of the final surgery examination.

#### Trial procedure

Each student assessed 11 AFs (3 anterior column fractures, 3 anterior column/wall and posterior hemitransverse fractures, 2 transverse fractures, 2 both-column fractures, 1 transverse and posterior wall fracture) presented in a fixed order using either the 3D tool or VR simulator. No immediate feedback was given; overall results were shown after all cases, with no time limit. Participants then completed a questionnaire on usability and experience. Demographic data (age, gender, semester, visualization type) were collected. Responses on seven items—as prior knowledge, runtime performance, intuitiveness, fracture identification, self-assessed accuracy, surgical interest, and gaming experience—were rated on a 5-point Likert scale.

#### Statistical analysis

Data were analyzed using IBM SPSS Statistics (v20, IMB Corp., Armonk, NY, USA). Classification accuracy was assessed for all, as well as essential and associated fractures seperately. Normality was tested with the Shapiro–Wilk test. The Mann–Whitney *U* test was used for comparison of both visualization tools (unpaired), whereas the Wilcoxon test was used for comparison of essential and associated fractures (paired). Significance was set at *p* < 0.05 with 95% confidence intervals. Subgroup analyses included 80 samples due to three missing detailed VR results; no imputation was performed. Questionnaire responses were analyzed descriptively using explorative data analysis. The number of valid responses differed between items due to occasional missing data.

## Results

Overall, 83 participants, 44 in the VR group and 39 in the 3D group, completed the study. There were 25 male participants (32%) and 54 female participants (68%). There were no significant sex differences in the two groups (*p* = 0.349). The median age of the participants was 24 years (IQR: 23–27) in the VR group and 25 years in the 3D group (IQR: 24–27).

### Classification

**Fig. 2:**
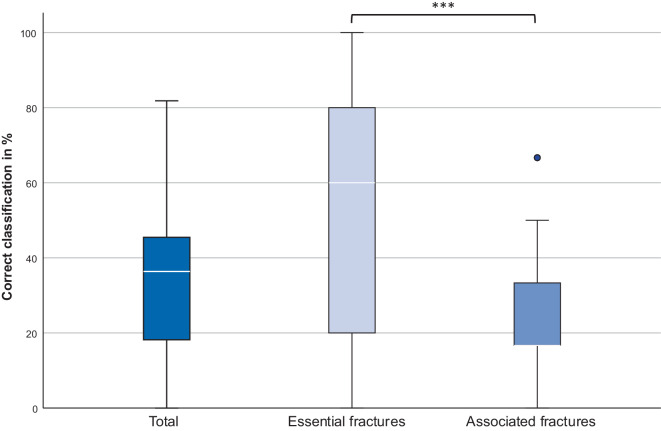
Percentage of correctly classified acetabular fractures across all participants regardless of the visualization tool. Essential fractures were classified significantly more correctly. Points beyond the whiskers represent outliers

In general, essential AFs were classified significantly more accurately than associated fractures (*p* < 0.001). The median score for essential fractures was 60% (IQR: 20–80%) and 16.6% (IQR: 17–33%) for associated fractures. In total, 36% of fractures (IQR: 18–45%) were classified correctly, regardless of visualization type (Fig. 2).

**Fig. 3:**
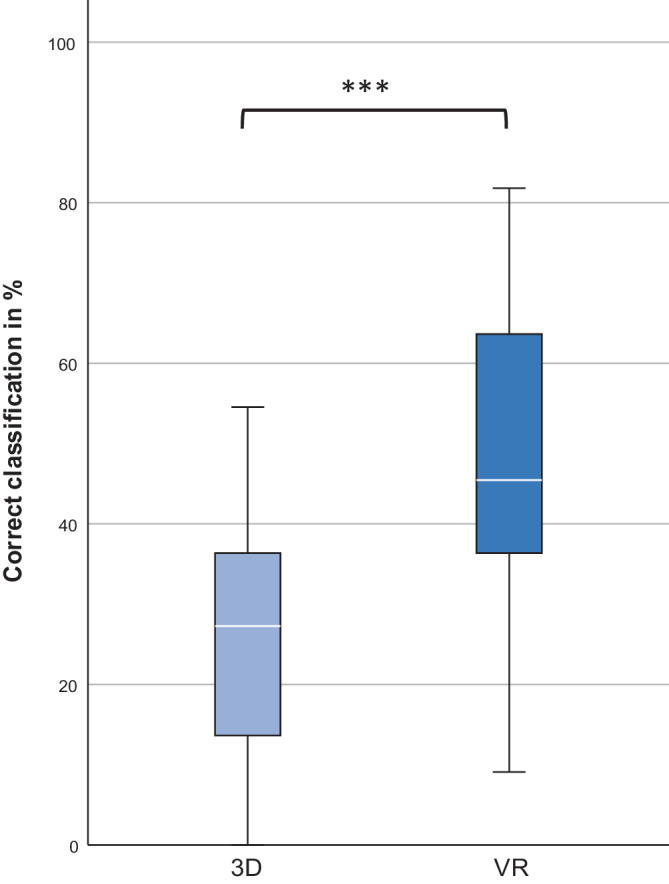
Percentage of correctly classified acetabular fractures in both visualization groups. The virtual reality (*VR*) group achieved significantly better results

The VR group achieved significantly higher classification accuracy (*p* < 0.001). In the control group, a median of 27% (IQR: 14–36%) of fractures were classified correctly, whereas the VR group achieved a median of 45% (IQR: 36–64%; Fig. 3).

**Fig. 4:**
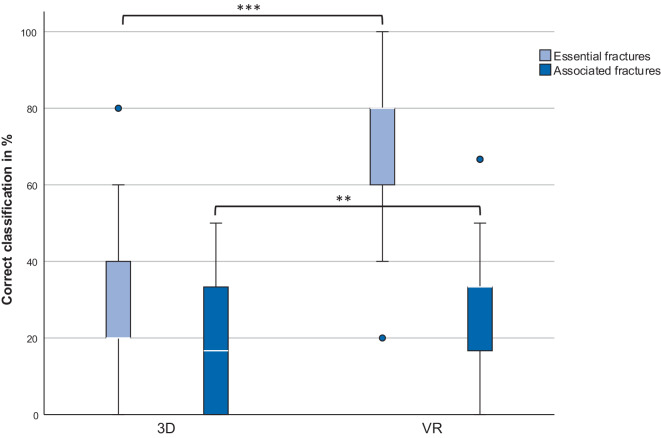
Comparison of essential and associated fractures for both groups. Both fracture types were classified significantly more correctly using virtual reality (*VR*) than three-dimensional (*3D*) visualization. Outliers are shown as individual points

Essential and associated fractures were classified more correctly by the VR group. Essential fractures were classified correctly in 20% (IQR: 20–40%) of cases by the 3D group and in 80% (IQR: 60–80%) of cases by the VR group (*p* < 0.001). The 3D group correctly classified a median of 17% (IQR: 0–33%) of associated fractures, whereas the VR group correctly classified a median of 33% (IQR: 17–33%). The difference was significant (*p* = 0.008; Fig. 4). Detailed results are presented in Table 1.

**Table 1:** Summary of classification results for both groups (3D vs. VR)

	Both groups	3D	VR	3D/VR
Total	**36%** (IQR 18–45%)	**27%** (IQR 14–36%)	**45%** (IQR 36–64%)	*p* *<* *0.001*
Essential fractures	**60%** (IQR 20–80%)	**20%** (IQR 20–40%)	**80%** (IQR 60–80%)	*p* *<* *0.001*
Associated fractures	**16.6%** (IQR 17–33%)	**17%** (IQR 0–33%)	**33%** (IQR 17–33%)	*p* *=* *0.008*
*Essential/associated*	*p* *<* *0.001*			

### Questionnaires

Of the 83 participants, 79 questionnaires were collected. All statements were rated on a 5-point Likert scale (1 = *strongly disagree*, 5 = *strongly agree*).

Most participants strongly agreed with the statement that acetabular surgery was largely unfamiliar to them (median = 5 [IQR: 4–5], *p* = 0.679). Regarding self-assessed quiz performance, the VR group rated themselves significantly higher (median = 3 [IQR: 1–5]) compared with the 3D group (median = 2 [IQR: 1–3], *p* = 0.017). The VR application was perceived as more intuitive to use (median = 5 [IQR: 4–5] vs. 4 [IQR: 3–5], *p* = 0.025), whereas the 3D application was perceived as having superior runtime performance (median = 5 [IQR: 4–5] vs. 4 [IQR: 4–5], *p* = 0.005).

**Fig. 5:**
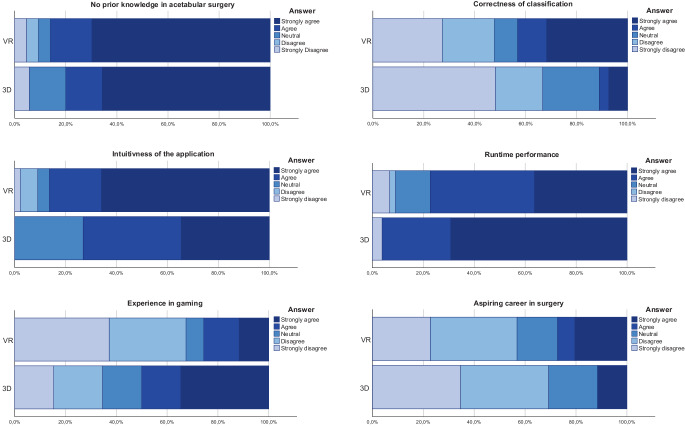
Distribution of answers for all questions in the questionnaire for the virtual reality (*VR*) and the three-dimensional (*3D*) group

Participants in the 3D group reported significantly more experience with computer games (median = 3.5 [IQR: 2–5]) compared to those in the VR group (median = 2 [IQR: 1–3.5], *p* = 0.007). Regarding the intention to pursue a surgical career, groups did not differentiate (3D: median = 2 [IQR: 1–3]; VR: median = 2 [IQR: 2–4], *p* = 0.171; Fig. 5).

## Discussion

As AFs have become more frequent and surgical training opportunities more limited, VR has emerged as a promising tool to enhance education. This study shows that immersive VR training can significantly improve AF classification accuracy among inexperienced users compared to conventional 3D visualization. Virtual reality offers a valuable supplement to traditional teaching in trauma surgery, especially for the challenging Letournel classification, which is essential for guiding treatment but remains difficult for novices and experts using standard radiographs or CT scans [1, 4, 20–22].

Considering that more than two-thirds of participants had no prior knowledge of AFs, the performance of the VR group, scoring up to 80% for essential fractures, demonstrates the potential of VR in teaching. Previous studies have shown that inexperienced users only achieve limited agreement when classifying AFs using conventional 3D visualization or CT viewers [23, 24]. The improvement can be attributed to the immersive nature of VR, which enhances spatial understanding and depth perception, allowing the users to intuitively explore anatomical structures without the restrictions of fixed rotational axes.

Patient safety is influenced by the correct treatment and surgical procedure, which has been shown to be improved directly by simulation-based training, enhancing communication and procedural performance in critical situations [21, 25]. Most research in surgical education has shown that immersive VR improves procedural accuracy and skill acquisition across multiple specialties [14, 16, 26]. However, most studies focused on psychomotor skills rather than cognitive tasks. Our results extend these findings by demonstrating that VR can also enhance diagnostic and analytical competencies, particularly spatial reasoning and fracture pattern identification, which are essential precursors to surgical decision-making. Whether the cognitive gains observed in VR translate into improved intraoperative spatial orientation and surgical planning remains to be investigated.

Integrating VR modules into the surgical curriculum can standardize exposure to rare and associated fractures, giving all trainees similar experience. Repetitive, patient-independent VR training may accelerate learning and support earlier independent decision-making, as shown in previous studies, which could potentially reduce individual workload [14].

The study also demonstrated that both groups estimated their performance realistically. The VR group self-assessed their personal work better than the 3D group did, concurring with the actual performance. Self-assessment is an important competency in medical practice. A reasonable self-assessment was shown to increase not only motivation, communication, and performance, but also patient safety [27]. Nevertheless, simulation training was shown to possibly induce overconfidence, and therefore personal skills need to be evaluated before taking responsibilities in clinical practice [28].

Virtual reality was rated as being significantly more intuitive, even among users with more limited gaming experience, indicating that easy usability for all users is feasible. Additionally, first usage could be simplified by integrating a structured tutorial, making personal instruction dispensable. Compared to the 3D application, the runtime performance of the VR tool was evaluated as being inferior, which can be attributed to software or hardware issues. This should be addressed in future versions because sluggish execution can be seen as one of the main factors for cybersickness [29].

Simulation tools offer cost-efficient training, as they involve minimal operating expenses once implemented. Despite initial hardware costs, benefits in procedural quality, patient safety, and complication reduction may outweigh the investment [15, 30].

### Limitations and challenges

Several limitations must be considered. Questionnaires from four VR participants were missing due to organizational issues, and the absence of time tracking precluded analysis of decision-making intensity. Learning and order effects may have occurred, as fractures were presented in a fixed sequence. External validity is limited by heterogeneous prior experience, and findings from medical students cannot be generalized to residents or clinical practice. Broader implementation would require integration into residency training and a permanent setup with staff for installation and instruction to ensure accessibility and feasibility.

### Outlook

Future studies should assess whether VR-based classification accuracy translates into clinical performance and outcomes. The modular platform enables expansion to additional fracture types, imaging integration, multi-user functionality, and augmented reality or haptic feedback to support cognitive, motor, and team-based training.

## Conclusion

Immersive virtual reality training improves the accuracy of acetabular fracture classification among less experienced users by enhancing spatial understanding. Its integration into surgical education can standardize training, accelerate skill acquisition, and potentially improve patient outcomes. Further studies should explore its effects on real-world clinical decision-making and wider orthopedic applications.

## Instructions for practice

- Integrate immersive VR training into surgical education: VR significantly enhances the accuracy of acetabular fracture classification, especially for inexperienced users.
- Offer regular VR sessions to foster spatial awareness and self-assessment; this supports safety and autonomy in daily clinical practice.
- Include structured VR modules in curricular training programs to provide standardized learning experiences and repeatable, patient-independent practice.
- Establish a permanent, easily accessible VR setup and provide tutorials for broad acceptance, ensuring no prior knowledge is needed.
- Use VR simulation specifically for training challenging fracture classifications to improve clinical decision-making and patient safety.

## Data Availability

The datasets can be requested in anonymized form from the corresponding author upon reasonable request.

## Declarations

No formal ethical approval was required, as the study involved voluntary participation in an educational exercise. Only anonymized age data were collected. All participants gave informed consent to participate in the study.
